# Nitric Oxide-Related Biological Pathways in Patients with Major Depression

**DOI:** 10.1371/journal.pone.0143397

**Published:** 2015-11-18

**Authors:** Andreas Baranyi, Omid Amouzadeh-Ghadikolai, Hans-Bernd Rothenhäusler, Simon Theokas, Christoph Robier, Maria Baranyi, Michael Koppitz, Gerhard Reicht, Peter Hlade, Andreas Meinitzer

**Affiliations:** 1 Department of Psychiatry, Medical University of Graz, Graz, Austria; 2 Institute for International Management Practice at ARU Cambridge, Cambridge, United Kingdom; 3 Hospital of the Brothers of St. John of God, Graz, Austria; 4 Clinical Institute of Medical and Chemical Laboratory Diagnostics, Medical University of Graz, Graz, Austria; Albany Medical College, UNITED STATES

## Abstract

**Background:**

Major depression is a well-known risk factor for cardiovascular diseases and increased mortality following myocardial infarction. However, biomarkers of depression and increased cardiovascular risk are still missing. The aim of this prospective study was to evaluate, whether nitric-oxide (NO) related factors for endothelial dysfunction, such as global arginine bioavailability, arginase activity, L-arginine/ADMA ratio and the arginine metabolites asymmetric dimethylarginine (ADMA) and symmetric dimethylarginine (SDMA) might be biomarkers for depression-induced cardiovascular risk.

**Methods:**

In 71 in-patients with major depression and 48 healthy controls the Global Arginine Bioavailability Ratio (GABR), arginase activity (arginine/ornithine ratio), the L-arginine/ADMA ratio, ADMA, and SDMA were determined by high-pressure liquid chromatography. Psychiatric and laboratory assessments were obtained at baseline at the time of in-patient admittance and at the time of hospital discharge.

**Results:**

The ADMA concentrations in patients with major depression were significantly elevated and the SDMA concentrations were significantly decreased in comparison with the healthy controls. Even after a first improvement of depression, ADMA and SDMA levels remained nearly unchanged. In addition, after a first improvement of depression at the time of hospital discharge, a significant decrease in arginase activity, an increased L-arginine/ADMA ratio and a trend for increased global arginine bioavailability were observed.

**Conclusions:**

Our study results are evidence that in patients with major depression ADMA and SDMA might be biomarkers to indicate an increased cardiovascular threat due to depression-triggered NO reduction. GABR, the L-arginine/ADMA ratio and arginase activity might be indicators of therapy success and increased NO production after remission.

## Introduction

Suffering from a depressive disorder is a prominent risk factor for myocardial ischemia and increased mortality following myocardial infarction [1–3]. In addition, Stewart et al. [4] reported that the relationship between depressive symptomatology and coronary artery calcification is driven largely by the affect cluster. However, biomarkers of depression and increased cardiovascular risk during depressive episodes have not been identified to date.

Independently from the mental state of a person, endothelial dysfunction is associated with increased cardiovascular risk and is defined by the reduced bioavailability of nitric oxide (NO), which plays an important role in flow-mediated vasodilatation [5,6]. In addition, NO inhibits aggregation of platelets, adhesion of monocytes and leukocytes to the endothelium, smooth muscle cell proliferation, oxidation of low density lipoprotein (LDL), and vascular inflammation by suppressing the expression and activity of chemokines and adhesion molecules [7–10]. The substrate for NO synthesis is arginine, which is converted to NO and citrulline by three nitric oxide synthase (NOS) isoforms: endothelial nitric oxide synthase (eNOS), inducible nitric oxide synthase (iNOS) and neuronal nitric oxide synthase (nNOS). The enzyme arginase competes with NOS and metabolizes l-arginine to ornithine [11]. Arginase activity is upregulated in several disease conditions such as diabetes mellitus or inflammation [11,12]. The l-arginine/ornithine ratio reflects arginase activity [13].

The newly introduced Global Arginine Bioavailability Ratio (GABR) might be a measure of endothelial function, because it accounts not only for the substrate l-arginine, but also for ornithine and citrulline and thus improves the poor prognostic value of l-arginine levels alone. GABR is calculated by dividing l-arginine by citrulline and ornithine [13]. Tang et al. [14] reported an association of GABR with significantly obstructive coronary artery disease and with future cardiovascular events in a large number of somatic patients without psychiatric disorders.

In summary, the effective production of NO is determined by the expression of nitric oxide synthase (NOS), arginase activity, the ratios of the reacting amino acids, the activity of enzymes with the same substrates, and the extent of attendant NOS inhibitors [13,14]. Asymmetric dimethylarginine (ADMA) is such an endogenous competitive inhibitor of NOS, and thus plays an important role as a regulator of NO production in the endothelium [15]. Symmetric dimethylarginine (SDMA) is a stereoisomer of ADMA and has no inhibitory effects on nitric oxide synthase. However, it may interfere with NO synthesis by competing with L-arginine for transport across cell membranes. The human liver seems to be a key organ in regulating ADMA and SDMA plasma concentrations [16]. As well as by renal elimination, ADMA is degraded by the two dimethylarginine dimethylaminohydrolases DDAH I and DDAH II to L-citrulline and dimethylamine. A lower L-arginine/ADMA ratio indicates less available NO [15,16,17]. S1 Fig shows the L-arginine and NO-Bioavailability.

### Aims of the Study

The direct measurement of NOS activity and NO is possible in vitro. In vivo these measures are more complex and difficult, but NOS activity and NO can be described regarding the reaction conditions. Thus, recent research studies assumed that the arginine bioavailability ratios (GABR and l-arginine/ornithine ratio), the L-arginine/ADMA ratio, the endogenous NOS inhibitor asymmetric dimethylarginine (ADMA) and its structural isomer symmetric dimethylarginine (SDMA) particularly impact on NO production in humans [13,18]. Consequently, the aim of this research project was to evaluate whether global arginine bioavailability, arginase activity, L-arginine/ADMA ratio and the l-arginine metabolites ADMA and SDMA are altered in patients with major depression and might thus be biomarkers for major depression-induced increased cardiovascular risk.

## Materials and Methods

### Participants

122 participants were recruited in this prospective study. Out of these participants 74 suffered from a major depression. The patients with depression were all in-patients, treated in the Department of Psychiatry, Hospital of the Brothers of St. John of God, Graz, Austria.

Three patients refused to participate in the study. Consequently, the final study sample consisted of 71 depressive patients and 48 healthy participants without depressive symptomatology and a former history of psychiatric disorders. Healthy controls were recruited using flyers placed throughout the city of Graz. The flyers briefly introduced the study and directed individuals interested in participating to contact study staff.

The following reasons for exclusion from enrolment applied to all study participants: (1) pregnancy, (2) significant co-morbid conditions (e.g. cardio-vascular illness, cancer), (3) disease or drugs that influence the immune system, (4) signs of infection and (5) diagnosis of a neurological disease.

In patients with major depression all psychiatric and biological assessments were carried out at two different times: at the time of in-patient admittance to a psychiatric hospital due to marked depressive symptomatology, and at the time of hospital discharge. The study has been approved by the Institutional Review Board of the University of Medicine of Graz. Data protection met the standards set by Austrian law. All participants in this study had to give signed informed consent, and subjects could decide to withdraw from this study at any time. The presented study has been carried out according to the GCP standards and the Declaration of Helsinki.

### Biological Assessments

Blood was sampled from the fasting subjects with major depression between 08.00 and 09.00 at baseline at the time of in-patient admittance to a Department of Psychiatry due to major depression symptomatology and at the time of hospital discharge for the assay of Global Arginine Bioavailability Ratio (GABR: l-arginine/[citrulline+ornithine]), arginase activity (l-arginine/ornithine), L-arginine/ADMA ratio, ADMA and SDMA. The same biological assessments were carried out in the healthy participants without a depressive symptomatology. From each subject, 16.5 mL of blood were drawn after short tourniquet using a 21-gauge needle into serum- (7.5 mL) and EDTA (9 mL) sample tubes (Sarstedt, Nuembrecht, Germany). The serum tubes were allowed to clot before centrifugation. Centrifugation was performed for 6 minutes at 4000 g.

ADMA and SDMA were measured in frozen EDTA plasma by high-performance liquid chromatography (HPLC) with solid phase extraction and precolumn derivatization first described by Teerlink et al. [19], with slight modifications [20]. Amino acids (l-arginine, ornithine and citrulline) were measured with modifications of previous described chromatographic methods (Roth [21], Schwarz et al. [22]). Briefly, after precipitation of EDTA plasma with perchloric acid following neutralization of the supernatant with sodium carbonate, the extracted amino acids were derivatized with o-phtalaldehyde and separated on a reversed phase column with gradient elution. Quantification were performed with ratios of fluorescence signals of the interesting amino acids to the internal standard norvaline in comparison to the appropriated calibration curves. Intra-assay and interassay CVs were all below 10%.

### Psychiatric Assessments

At every time of examination all participants were evaluated by means of an author-compiled sociodemographic questionnaire, a clinical interview, the observer-rating Hamilton Depression Scale (HAMD-17, Hamilton [23]) and the self-rating Beck Depression Inventory scale (BDI-II, Beck et al. [24]) by experienced psychiatrists (O. A.-G., A. B.).

1. **Author-compiled sociodemographic questionnaire.** Sociodemographic variables of the author-compiled questionnaire included age, gender, living arrangements, employment status and marital status at the time of the psychiatric assessment. Marital status has been categorized as single, partner, widowed, and divorced.
2. **Hamilton Depression Scale (HAMD-17, Hamilton [23]).** The 17-item Hamilton rating scale for depression (HAMD-17) is used to measure the severity of depressive symptomatology. A score of 18 points or more typically equals major depression, the range of 14–17 points generally indicates moderate depressive symptomatology, and the range of 10–13 points indicates mild depressive symptomatology [23].
3. **Beck Depression Inventory (BDI-II, Beck et al. [24]).** The Beck Depression Inventory (BDI) is a 21-question multiple-choice survey and one of the most frequently used instruments for measuring the severity of depression. The questionnaire consists of items relating to depression symptoms such as hopelessness and irritability, cognitions such as guilt or feelings of being punished, as well as physical symptoms such as fatigue, weight loss, and lack of interest in sex. The total values of the BDI-II can range between 0 and 63 points. Values under 10 are considered as insignificant [24].

### Statistical Analyses

Descriptive statistics were produced based on demographic, treatment-related, biochemical and psychometric data and are presented as mean and standard deviation (SD). Chi-square tests were used to evaluate group differences in categorical variables. ADMA and citrulline were normally distributed, as shown by the Shapiro-Wilk test. In addition, equal variances were determined for those parameters. Consequential for these parameters, differences between groups were assessed using t-tests. HAMD-17 and BDI-II scores, SDMA, GABR, the l-arginine/ornithine ratio, l-arginine and ornithine were not normally distributed. We therefore applied the non-parametric Mann-Whitney U test, or where appropriate, the Wilcoxon test. All statistical tests were two-tailed, with significance set at p<0.05. Due to the exploratory character of this study the results were not α adjusted for multiplicity. All statistical analyses were performed with the R Project for Statistical Computing (R Development Core Team, 2011) and SPSS 18.0 for Windows (SPSS; Chicago, IL).

## Results

### Sociodemographic and Treatment Characteristics

All participating 71 patients with major depression and the 47 healthy controls were Caucasian. At the time of in-patient admittance to the Department of Psychiatry the patients with major depression had a mean HAMD-17 score of 21.1 (SD = 4.9) and a mean BDI-II score of 24.5 (SD = 9.7).

After an average hospital stay of 16.1 days a significant psychic improvement at the time of discharge was observed [mean HAMD-17 score at the time of discharge: 9.7 (SD = 6.7), mean BDI-II score at the time of discharge 14.8 (SD = 11.5)]. 70 patients had a medication-based therapy with selective serotonin re-uptake inhibitors or second-generation dual-action antidepressants. One patient had a medication-based therapy with a tricyclic antidepressant.

Table 1 summarizes the sociodemographic characteristics for the whole sample and the clinical characteristics for the subsample of participants with major depression at the time of in-patient admittance.

**Table 1:** Sociodemographic and clinical characteristics.

Category	Major Depression (n = 71)	Healthy Controls (n = 48)	p
**Gender**			
Male	48 (67.6%)	31 (64.6%)	χ^2^ = 0.117; df = 1; p = 0.732^a^
Female	23 (32.4%)	17 (35.4%)	
**Age**			
Median	50	40	Mann-Whitney-U-Test: 1423.0, p = 0.13^b^
25^th^ percentile	43.0	30.8	
75^th^ percentile	56.0	66.8	
**Marital status**			
Single	16 (23.2 %)	17 (35.4 %)	χ^2^ = 2.608; df = 3; p = 0.456^a^
Partner	48 (69.6 %)	28 (58.3 %)	
Widowed	2 (2.9 %)	2 (4.2 %)	
Divorced	3 (4.3 %)	1 (2.1 %)	
**Employment status**			
Paid work	32 (46.4 %)	23 (47.9 %)	χ^2^ = 0.27; df = 1; p = 0.870^a^
No paid work (homeworker, un-employed, retired)	37 (53.6 %)	25 (52.1 %)	
**Living arrangements**			
Alone	18 (26.1%)	11 (22.9%)	χ^2^ = 0.153; df = 1; p = 0.696^a^
With others (family, partner or friends)	51 (73.9%)	37 (77.1%)	
**Systolic blood pressure**			
Mean	123.6	120.9	t = 1.0, df = 98; p = 0.320^c^
SD	1.9	1.7	
**Diastolic blood pressure**			
Median	80.0	78.0	Mann-Whitney-U-Test: 1012.50, p = 0.155 ^b^
25^th^ percentile	70.0	70.5	
75^th^ percentile	80.0	80.0	
**Body height**			
Median	171.0	168.0	Mann-Whitney-U-Test: 1087.0, p = 0.032 ^b^
25^th^ percentile	165.0	162.0	
75^th^ percentile	180.0	172.0	
**Bodyweight**			
Median	74.0	69.0	Mann-Whitney-U-Test: 1193.0, p = 0.136 ^b^
25^th^ percentile	66.5	58.0	
75^th^ percentile	90.65	85.0	
**BMI**			
Median	26.2	22.9	Mann-Whitney-U-Test: 916.0, p = 0.001 ^b^
25^th^ percentile	24.3	21.0	
75^th^ percentile	32.4	28.1	

Legend:

SD = Standard deviation

^a^ χ^2^ tests.

^b^ Mann-Whitney-U-Test

^c^ t-Test

Table 2 shows the HAMD-17 and BDI-II scores for the participants with major depression and the healthy controls.

**Table 2:** Biological assessments and psychometric tests for the patients with major depression at the time of inpatient admittance, at the time of hospital discharge and for the healthy controls.

	I: Healthy Controls (n = 48)	II: Patients with major depression at the time of inpatient admittance (n = 71)	III: Patients with major depression at the time of of hospital discharge	p
**HAMD-17**	Median:0.000	Median: 20,000	Median: 10,000	I-II: Mann-Whitney-U-Test: 0.0; **p <0.0001** ^a^
	25^th^ percentile: .000	25^th^ percentile: 18,000	25^th^ percentile: 4,000	II-III: Wilcoxon Z: -6.657; **p <0.0001** ^b^
	75^th^ percentile: .000	75^th^ percentile: 24,000	75^th^ percentile: 14,000	
**BDI-II**	Median: 1.000	Median: 24.000	Median: 11.000	I-II: Mann-Whitney-U-Test: 9.5; **p <0.0001** ^a^
	25^th^ percentile: .000	25^th^ percentile: 17,250	25^th^ percentile: 6.000	II-III: Wilcoxon Z: -5.733; **p <0.0001** ^b^
	75^th^ percentile: 2.750	75^th^ percentile: 32.000	75^th^ percentile: 24.000	
**Global Arginine Bioavailability Ratio (GABR)**	Median: 0.833	Median: 0.858	Median: 0.920	I-II: Mann-Whitney-U-Test: 1561.5; p = 0.69^a^
	25^th^ percentile: 0.680	25^th^ percentile: 0.675	25^th^ percentile: 0.721	II-III: Wilcoxon Z: -1.814, **p = 0.070** ^b^
	75^th^ percentile: 0.999	75^th^ percentile: 1.023	75^th^ percentile: 1.090	
**Arginase Activity Ratio [L-Arginine/Ornithine]**	Median: 1.092	Median: 1.153	Median: 1.256	I-II: Mann-Whitney-U-Test: 1544.5; p = 0.621^a^
	25^th^ percentile: 0.882	25^th^ percentile: 0.929	25^th^ percentile: 0.920	II-III: Wilcoxon Z: -2.057; **p = 0.040** ^b^
	75^th^ percentile: 1,347	75^th^ percentile: 1.488	75^th^ percentile: 1.529	
L-Arginine, μmol/L	Median: 97.440	Median: 101.500	Median: 101.750	I-II: Mann-Whitney-U-Test: 1469.0; p = 0.36^a^
	25^th^ percentile: 86.117	25^th^ percentile: 86.190	25^th^ percentile: 85.790	II-III: Wilcoxon Z: -1.746; p = 0.081^b^
	75^th^ percentile: 104.697	75^th^ percentile: 112.940	75^th^ percentile: 117.980	
Ornithine, μmol/L	Median: 94.050	Median: 82.64	Median: 82.450	I-II: Mann-Whitney-U-Test: 1558.0; p = 0.431^a^
	25^th^ percentile: 72.550	0 25^th^ percentile: 70.390	25^th^ percentile: 74.450	II-III: Wilcoxon Z: -0.548; p = 0.584^b^
	75^th^ percentile: 103.775	75^th^ percentile: 101.290	75^th^ percentile: 100.170	
Citrulline, μmol/L	Mean: 30.648;	Mean: 29.887	Mean: 30.45	I-II: t = -0.597, df = 117;p = 0.552^c^
	SD = 7.63	SD = 6.23	SD = 6.48	II-III: t (paired) = -0.759, df = 62; p = 0.451^d^
**Asymmetric Dimethylarginine (ADMA), μmol/L**	Mean: 0.612	Mean: 0.645	Mean: 0.629	I-II: t = 2.052, df = 116; **p = 0.042** ^c^
	SD = 0.08	SD = 0.09	SD = 0.11	II-III: t (paired) = 1.057, df = 56; p = 0.295^d^
**Symmetric Dimethyl-arginine (SDMA), μmol/L**	Median: 0.705	Median: 0.660	Median: 0.630	I-II: Mann-Whitney-U-Test: 1172.5; **p = 0.005** ^a^
	25^th^ percentile: 0.612	25^th^ percentile: 0.560	25^th^ percentile: 0.525	II-III: Wilcoxon Z: -0.402; p = 0.688^b^
	75^th^ percentile: 0.810	75^th^ percentile: 0.712	75^th^ percentile: 0.725	
**L-Arginine/ADMA**	Mean: 160.314	Mean: 156.329	Mean: 166.505	I-II: t = -0.631 df = 114; p = 0.529^c^
	SD = 24.672	SD = 37.817	SD = 38.854	II-III: t (paired) = -2.257, df = 56; **p = 0**.**028** ^d^ ^:^

^a^ Mann-Whitney-U-Test

^b^ Wilcoxon signed rank Test

^c^ t-Test

^d^ Paired t-Test

### Global Arginine Bioavailability, Arginase Activity and L-Arginine/ADMA Ratio

**Fig 1:**
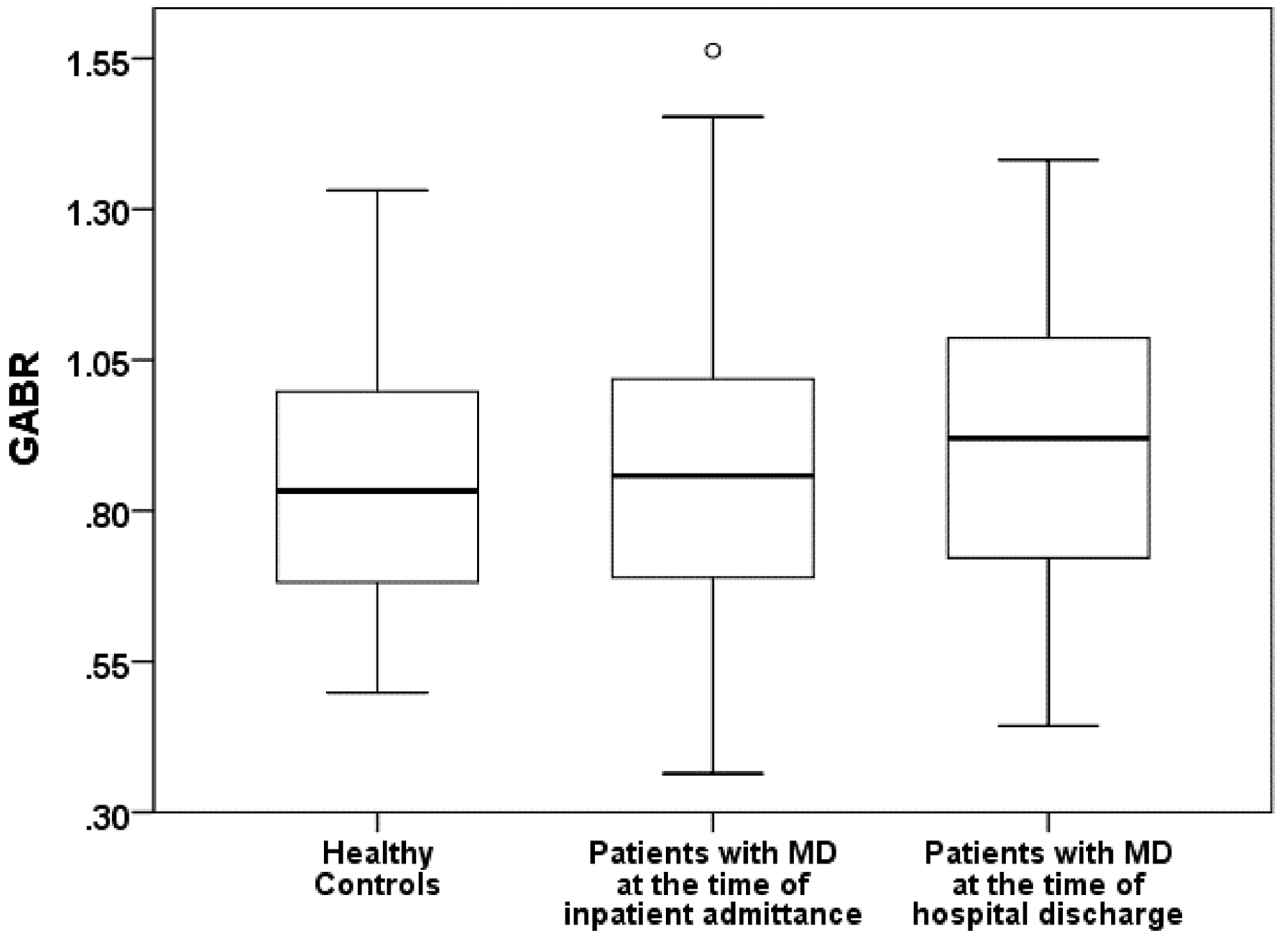
Comparison of the Global Arginine Bioavailability (GABR) between healthy contols and patients with major depression (MD).

**Fig 2:**
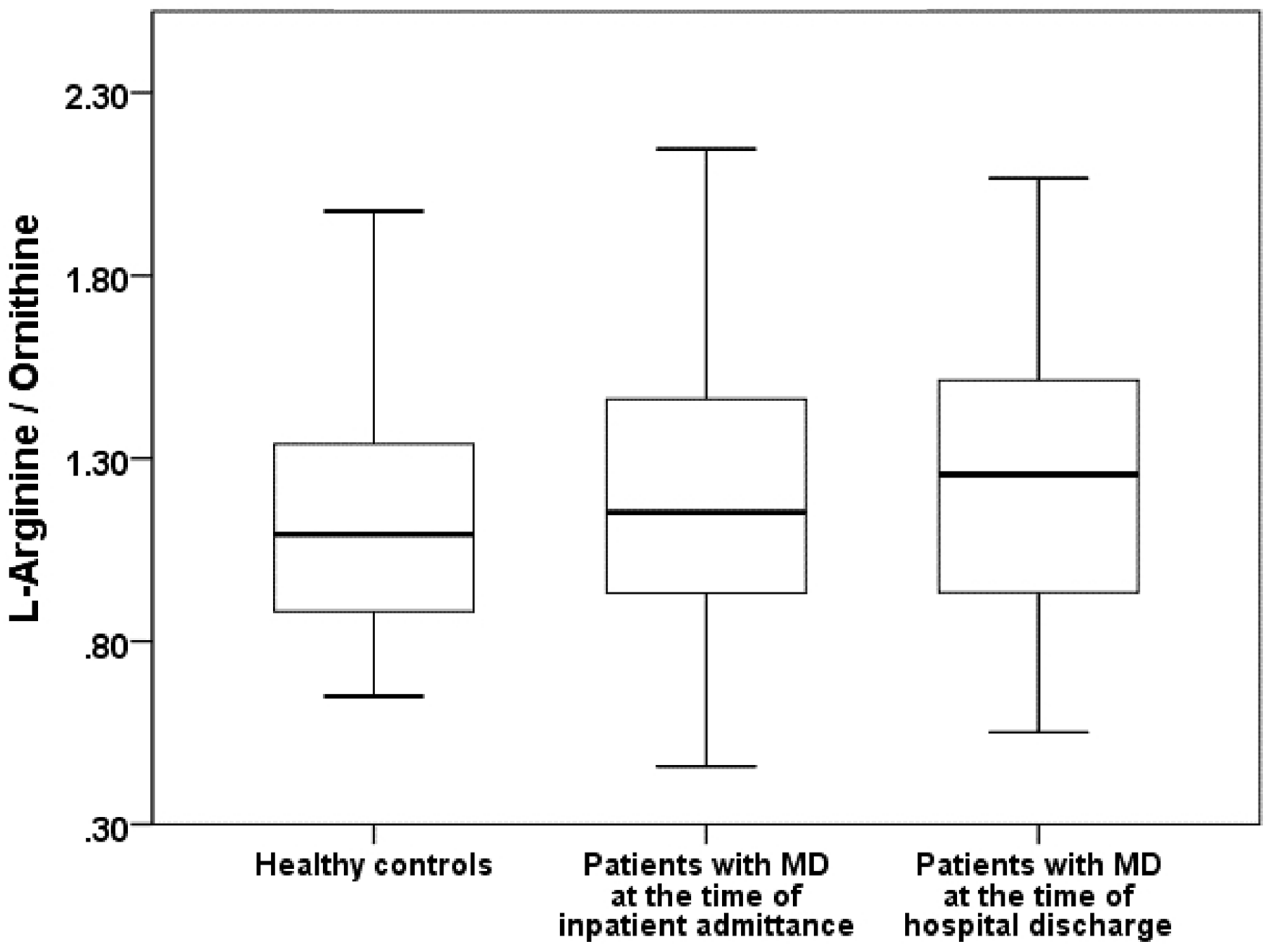
Comparison of the arginase activity ratio between healthy contols and patients with major depression (MD).

Primarily patients suffering from major depression had a global arginine bioavailability (GABR: Mann-Whitney-U: 1561.5; p = 0.69) **(** Fig 1), arginase activity (Mann-Whitney-U-Test: 1544.5; p = 0.621) (Fig 2) and L-arginine/ADMA ratio (t = -0.631 df = 114; p = 0.529) similar to those of healthy controls without a history of psychiatric disorders. After a first improvement of depression, a significant decrease in arginase activity (l-arginine to ornithine ratio ↑) in patients suffering from major depression was observed at the time of hospital discharge (Wilcoxon Test: Z = -2.057; p = 0.04) (Fig 2). In addition, there was a trend for increased global arginine bioavailability and a significant increase of the L-arginine/ADMA ratio in depressive patients at the time of hospital discharge (GABR: Wilcoxon Test: Z = -1.814; p = 0.07 (Fig 1); L-arginine/ADMA ratio: t (paired) = -2.257, df = 56; p = 0.028).

Table 2 summarizes the Global Arginine Bioavailability Ratio, arginase activity, the L-arginine/ADMA ratio, and l-arginine, ornithine and citrulline concentrations for the patients with major depression and for the healthy controls.

### Asymmetric Dimethylarginine (ADMA) and Symmetric Dimethylarginine (SDMA)

**Fig 3:**
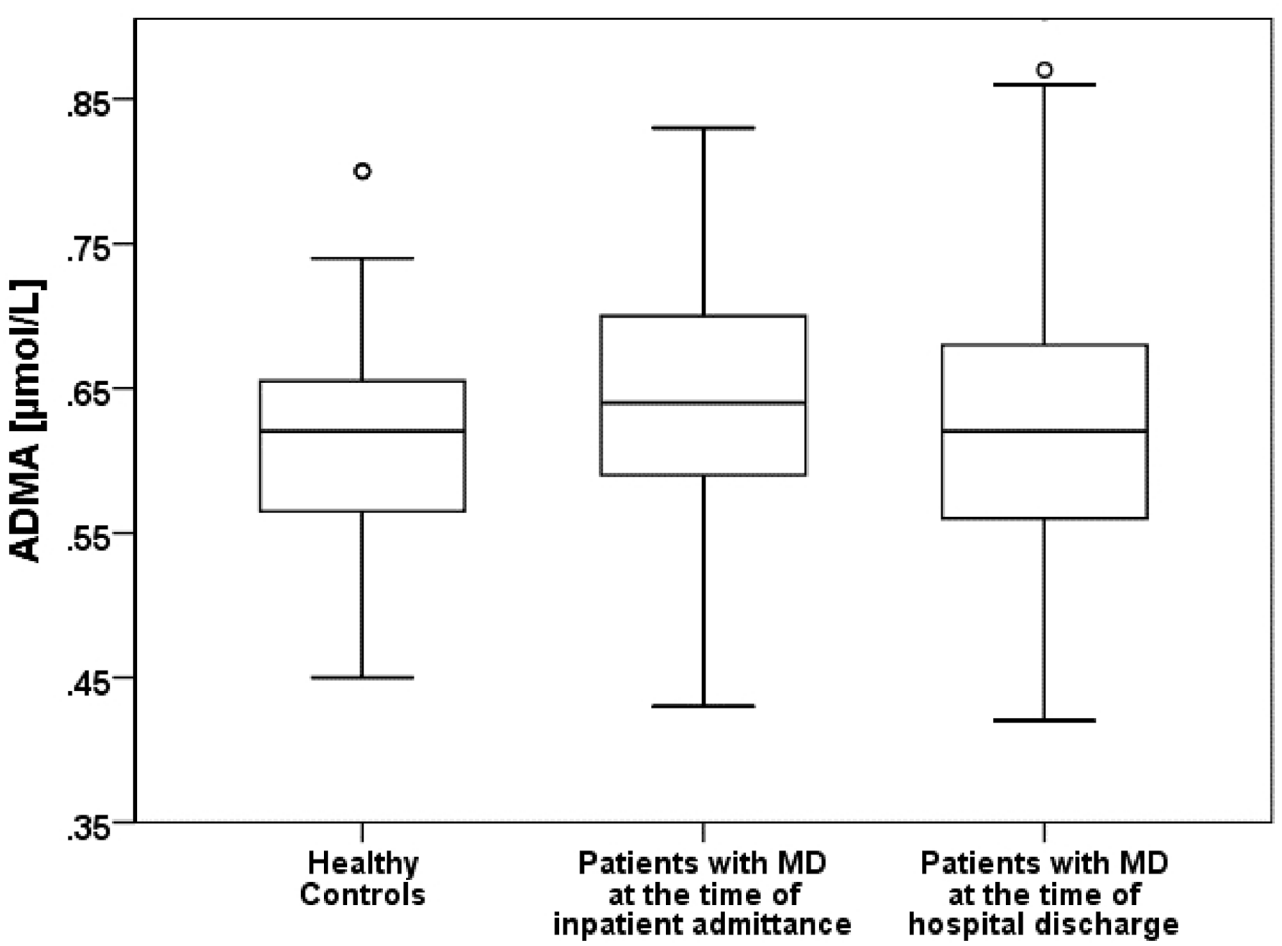
Comparison of Asymmetrical Dimethylarginine (ADMA) between healthy contols and patients with major depression (MD).

In patients with major depression the ADMA concentrations were significantly elevated in comparison with healthy participants (t = 2.052, df = 116; p = 0.042). Even after a short-term improvement of depression at the time of discharge from a psychiatric hospital, ADMA levels remained nearly unchanged (t (paired) = 1.057, df = 56; p = 0.295) (Fig 3).

**Fig 4:**
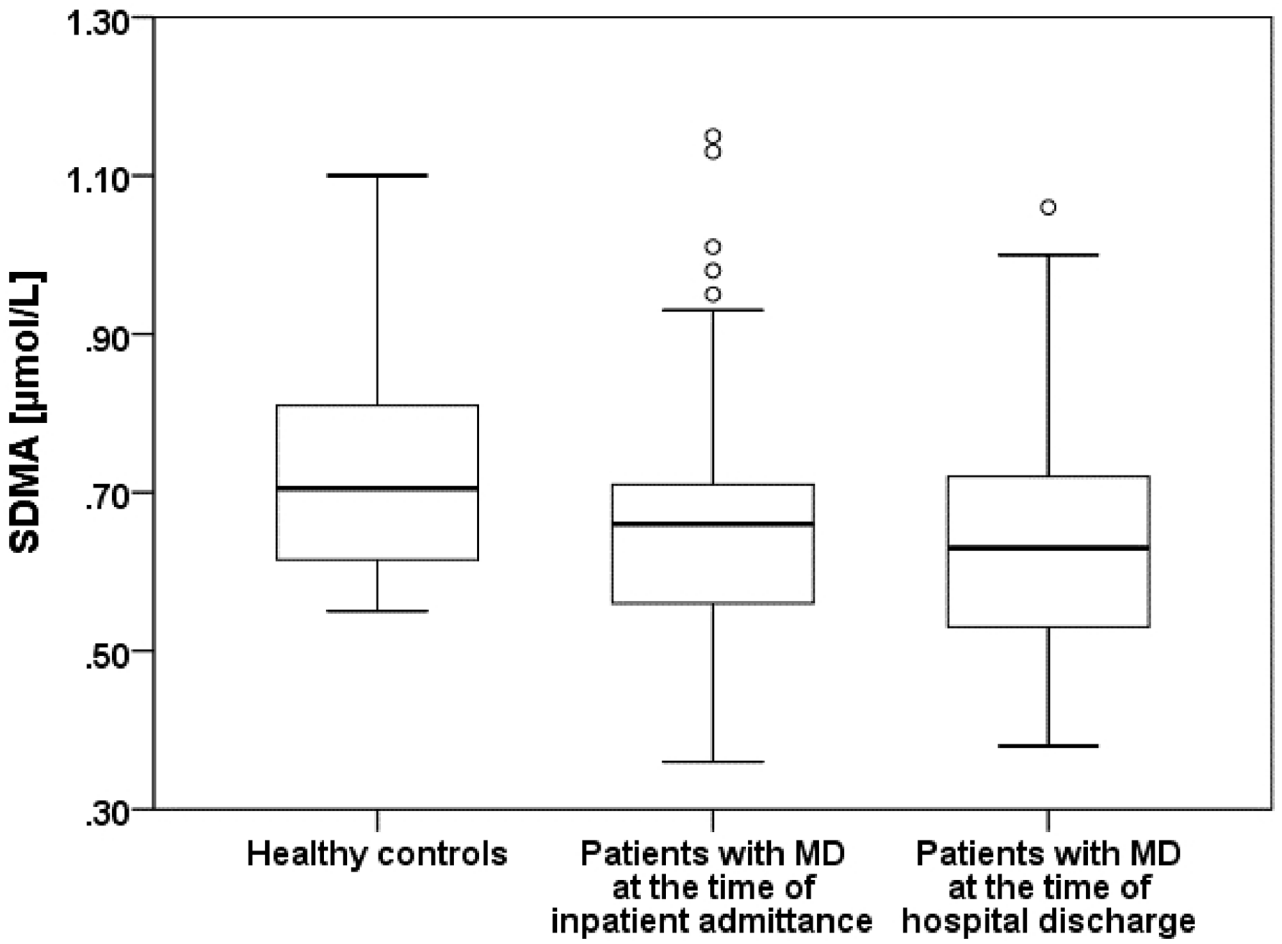
Comparison of Symmetrical Dimethylarginine (SDMA) between healthy contols and patients with major depression (MD).

SDMA concentrations were significantly lower in patients with major depression than those of healthy controls (Mann-Whitney-U-Test: 1172.5; p = 0.005) (Fig 4).

Table 2 shows the ADMA and SDMA concentrations for the patients suffering from major depression and for the healthy controls.

## Discussion

Endothelial dysfunction is associated with increased cardiovascular risk and defined by the reduced bioavailability of NO. The Global Arginine Bioavailability Ratio (GABR) accounts for l-arginine, ornithine and citrulline, and thus is an innovative ratio to measure endothelial dysfunction due to reduced bioavailability of NO. The l-arginine/ornithine ratio reflects arginase activity and is associated with markers of endothelial dysfunction and increased risk of cardiovascular mortality. In this study global arginine bioavailability and arginase activity were not primarily altered in patients suffering from major depression. However, after a first improvement of depression at the time of discharge from the psychiatric hospital, a significant decrease in arginase activity (l-arginine to ornithine ratio ↑) as well as a trend for increased global arginine bioavailability (GABR) were observed. Both described mechanisms particularly impact on NO production and thus might be indicators of therapy success. The effective production of NO is further determined by the extent of asymmetric dimethylarginine (ADMA), an endogenous competitive inhibitor of nitric oxide synthase in the endothelium [25]. The results of this prospective study demonstrate that in patients with major depression ADMA concentrations are significantly elevated in comparison with healthy subjects. Even after a short-term improvement of depression at the time of discharge from a psychiatric hospital ADMA levels remain nearly unchanged. This observation clearly suggests that there might be a prolonged elevation of ADMA in patients with major depression even after subjective improvement of depressive symptoms.

First indicators showing a possible association of ADMA and depression were introduced in the literature by Selley. [26]. In a previous study of our research group the associations of interferon-α (IFN-α) induced depressive symptoms with ADMA and SDMA in patients with chronic hepatitis C infection were prospectively investigated until three months post treatment [18]. During IFN-α treatment, ADMA concentrations increased significantly in patients suffering from IFN-α induced depression and returned to concentrations comparable to baseline values three months after the end of IFN-α treatment, suggesting a major impact of IFN-α and inflammatory system on ADMA concentrations. In this former study the increase of SDMA was not associated with treatment-induced depression. As a conclusion of our previous study we were able to show for the first time that depression in response to IFN-α treatment is associated with elevated ADMA levels. In addition, a study by Mommersteeg et al. [17] showed that in patients with heart failure, depressive symptoms were associated with markers of NO dysregulation, particularly the L-arginine/ADMA ratio. Depressive symptoms were correlated with a lower L-arginine/ADMA ratio and higher SDMA levels. A lower L-arginine/ADMA ratio indicates less available NO. In our study a significant higher L-arginine/ADMA ratio was observed after a first improvement of depression at the time of discharge from the psychiatric hospital.

There is growing evidence that ADMA might be a biomarker to indicate cardiovascular risk [27,28]. According to Meinitzer et al. [29] ADMA levels are generally not associated with the severity of a cardiovascular diseases as diagnosed e.g. with angiography. However, an elevated ADMA level might increase the risk of fatal events due to cardiovascular diseases [30]. In addition, elevated levels of ADMA in the pericardial fluid of cardiac patients correlate with cardiac hypertrophy [31].

Furthermore, in patients without coronary heart disease or a peripheral arterial occlusive disease, the plasma ADMA level is significantly correlated with risk factors of arteriosclerosis and with carotid intima-media thickness [32]. In addition, increased plasma concentrations of ADMA are associated with hypertriglyceridaemia, which may also cause endothelial dysfunction. Oxidised LDL, an important risk factor of arteriosclerosis, increases the production of ADMA by inhibiting dimethylaminohydrolase [33].

In depression the impact of ADMA may not only present an increased cardiovascular threat due to ADMA-triggered NO reduction, but the increased production of ADMA may also lead to a reduction in the amount of NO diffusing from the endothelium to neurons. This factor might negatively affect excitability and neurotransmission, mainly in the amygdala, the locus coeruleus, the hippocampus, the striatum, and the hypothalamus [34,35]. In this way, ADMA-related impaired neurotransmission might be an essential aggravating contributor to depressive symptomatology [25,34,36]. Recent studies have additionally demonstrated that the pharmacological inhibition of NO synthesis reduces spontaneous and stimulant-induced activity and is associated with anxiogenic effects [37]. In a study by Kielstein et al. [38] Brain-derived neurotrophic factor (BDNF) levels were inversely related to the degree of depression measured by Beck Depression Inventory. In rats, ADMA infusion led to significantly decreased BDNF levels. Within 4 weeks, 5/6 nephrectomy led to increased anxiety, decreased exploratory behavior and decreased spontaneous locomotion. In this study all of these effects could also be induced by long-term continuous infusion of ADMA to rats with normal renal function. In contrast, infusion of SDMA in mice did not exhibit any biological effects [38].

Even an association between increased ADMA concentrations and impaired cognitive function was observed in studies by Bajaj et al. [39] and Asif et al. [40].

Furthermore, ADMA influences cerebral circulation via NO and thus has a negative impact on cerebral ischemia or even stroke. It is well known that cerebral blood flow is decreased in many patients suffering from major depression, and this reduction might be mediated by increased ADMA concentrations during depressive episodes [41].

In a large prospective study (LURIC 1) with over 3000 participants no correlation between ADMA and the Body Mass Index (BMI) was observed [29,42].

The stereoisomer of ADMA is called symmetric dimethylarginine (SDMA), and has no known inhibitory effects on nitric oxide synthase. However, SDMA may interfere with NO synthesis by competing with L-arginine for transport across cell membranes. In the present study SDMA concentrations were significantly lower in patients with major depression than those of healthy participants without a history of psychiatric disorders. The decrease of SDMA in patients with major depression could potentially be attributed to different influences of depression in the synthesis of SDMA and ADMA. SDMA is synthesized by type II protein methyltransferase, while ADMA is mostly produced by methyltransferase of type I [43]. In patients with major depression these enzymes may be differently influenced. However, differences in clearance might also be possible, especially since SDMA is completely cleared by the kidney [43].

### Limitations

Further studies are required to determine any set patterns. All participants were instructed to fast for 10 hours before every blood sampling and all participants were strictly required to adhere to these instructions, or otherwise to step back from participation. However, it is not guaranteed that every participating patient adhered to this instruction. Further research may evaluate the impact of variables such as cholesterol/triglyceride levels, renal and liver function and injury markers. The cause of prolonged elevation of ADMA in patients with major depression even after subjective improvement of depressive symptoms requires further studies. Knowledge about the complex health consequences of nitric oxide-related biological pathways and cardiovascular risk during depression is still limited and requires ongoing research.

## Conclusions

Global arginine bioavailability, arginase activity, and the l-arginine metabolites ADMA and SDMA are well known biomarkers in somatic patients for indicating cardiovascular diseases. Our study results suggest that even in patients with major depression, ADMA and SDMA are altered compared to healthy subjects and thus may represent biomarkers that could indicate an increased cardiovascular threat due to depression-triggered NO reduction, while global arginine bioavailability, the L-arginine/ADMA ratio and arginase activity might be indicators of therapy success and increased NO production after remission.

In the future, ADMA and SDMA may contribute to identifying a subpopulation of depressed patients at greatest risk of cardiovascular disease in whom early intervention would be necessary, and risk reduction could be achieved with intervention specially designed to reduce depression-associated cardiovascular risk.

## Supporting Information

S1 Dataset (XLSX)

S1 Fig (PDF)

## Abbreviations

ADMA: asymmetric dimethylarginine
BDI: Beck Depression Inventory
BDNF: Brain-derived neurotrophic factor
DDAH I: dimethylarginine dimethylaminohydolase I
DDAH II: dimethylarginine dimethylaminohydolase II
eNOS: endothelial nitric oxide synthase
GABR: Global Arginine Bioavailability Ratio
HAMD-17: Hamilton Rating Scale for Depression
iNOS: inducible nitric oxide synthase
LDL: low density lipoprotein
NO: nitric oxide
nNOS: neuronal nitric oxide synthase
NOS: nitric oxide synthase
SD: standard deviation
SDMA: symmetric dimethylarginine
