# Supplementary figures and images for: Nitric Oxide-Related Biological Pathways in Patients with Major Depression
Source: PLoS One. 2015 Nov 18;10(11):e0143397. doi: 10.1371/journal.pone.0143397 (PMC4651499; doi:10.1371/journal.pone.0143397)

### S1 Fig. L-Arginine and NO-Bioavailability

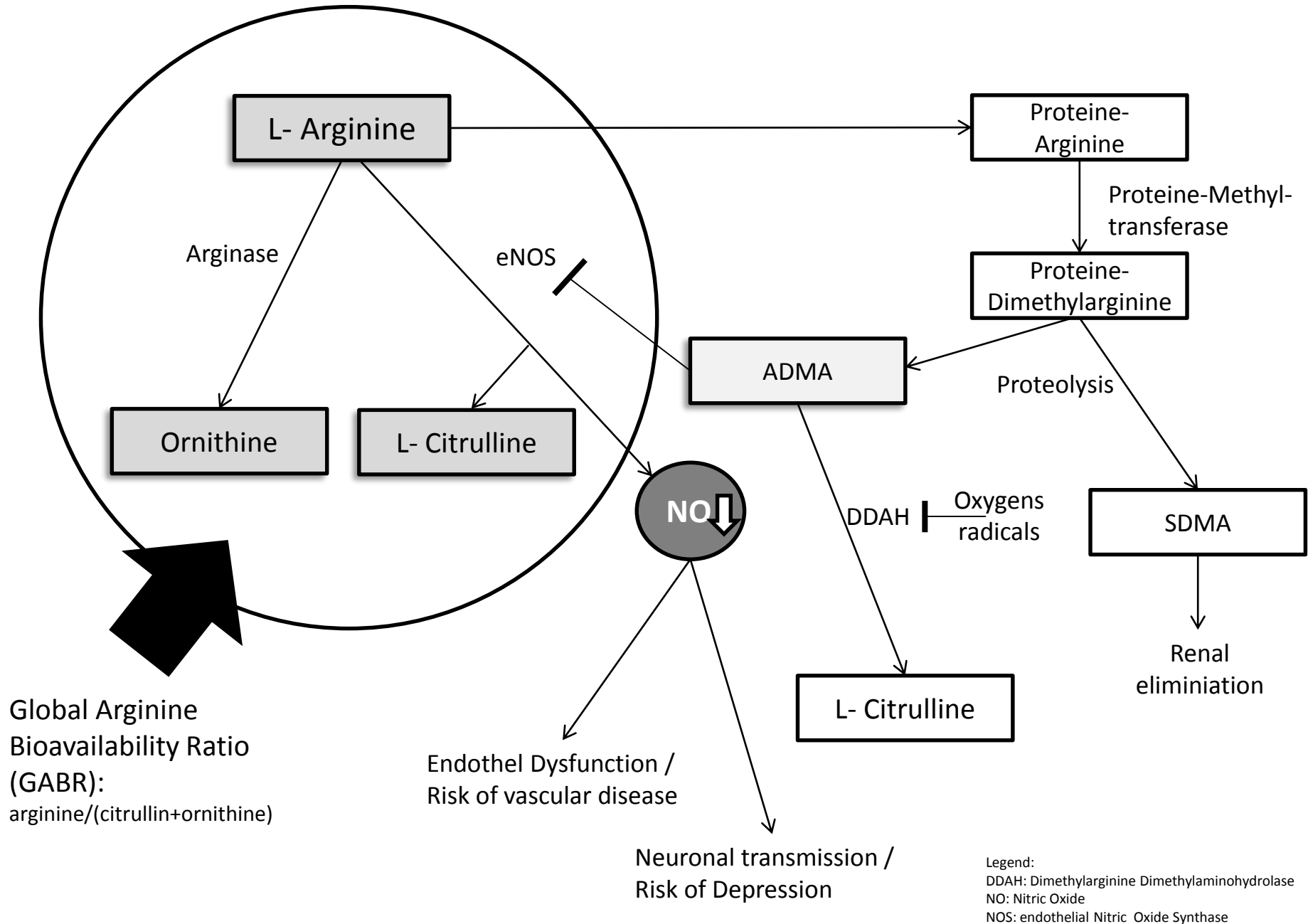

Supplement: S1 Fig — (PDF) [file pone.0143397.s002.pdf]
